# Decision aids linked to evidence summaries and clinical practice guidelines: results from user-testing in clinical encounters

**DOI:** 10.1186/s12911-021-01541-7

**Published:** 2021-06-29

**Authors:** Anja Fog Heen, Per Olav Vandvik, Linn Brandt, Frankie Achille, Gordon Henry Guyatt, Elie A. Akl, Shaun Treewek, Thomas Agoritsas

**Affiliations:** 1 Department of Medicine, Innlandet Hospital Trust, Mailbox 104, 2381 Brumunddal, Norway; 2 MAGIC Evidence Ecosystem Foundation, Oslo, Norway; 3 Institute for Health and Society, Faculty of Medicine, University of Oslo, Oslo, Norway; 4 Department of Clinical Epidemiology and Biostatistics, Faculty of Health Sciences, McMaster University, Hamilton, ON Canada; 5 Department of Internal Medicine, American University of Beirut, Beirut, Lebanon; 6 Health Services Research Unit, University of Aberdeen, Aberdeen, UK; 7 Division of Clinical Epidemiology and Division of General Internal Medicine, University Hospitals of Geneva, Geneva, Switzerland

**Keywords:** Decision aids, Shared decision-making, Clinical practice guidelines

## Abstract

**Background:**

Tools for shared decision-making (e.g. decision aids) are intended to support health care professionals and patients engaged in clinical encounters involving shared decision-making. However, decision aids are hard to produce, and onerous to update. Consequently, they often do not reflect best current evidence, and show limited uptake in practice. In response, we initiated the Sharing Evidence to Inform Treatment decisions (SHARE-IT) project. Our goal was to develop and refine a new generation of decision aids that are generically produced along digitally structured guidelines and evidence summaries.

**Methods:**

Applying principles of human-centred design and following the International Patient Decision Aid Standards (IPDAS) and GRADE methods for trustworthy evidence summaries we developed a decision aid prototype in collaboration with the Developing and Evaluating Communication strategies to support Informed Decisions and practice based on Evidence project (DECIDE). We iteratively user-tested the prototype in clinical consultations between clinicians and patients. Semi-structured interviews of participating clinicians and patients were conducted. Qualitative content analysis of both user-testing sessions and interviews was performed and results categorized according to a revised Morville’s framework of user-experience. We made it possible to produce, publish and use these decision aids in an electronic guideline authoring and publication platform (MAGICapp).

**Results:**

Direct observations and analysis of user-testing of 28 clinical consultations between physicians and patients informed four major iterations that addressed readability, understandability, usability and ways to cope with information overload. Participants reported that the tool supported natural flow of the conversation and induced a positive shift in consultation habits towards shared decision-making. We integrated the functionality of SHARE-IT decision aids in MAGICapp, which has since generated numerous decision aids.

**Conclusion:**

Our study provides a proof of concept that encounter decision aids can be generically produced from GRADE evidence summaries and clinical guidelines. Online authoring and publication platforms can help scale up production including continuous updating of electronic encounter decision aids, fully integrated with evidence summaries and clinical practice guidelines.

**Supplementary Information:**

The online version contains supplementary material available at 10.1186/s12911-021-01541-7.

## Background

Most medical decisions are highly context-dependant, and, when creating individual plans of care, current best evidence of potential benefits and harms requires interpretation in light of patients’ values and preferences. Shared decision-making is the process in which patients and clinicians partner together and have a conversation to find the best option for that patient [1]. Communicating evidence for shared decision-making is challenging [2]. Trustworthy clinical practice guidelines (henceforth guidelines) are amongst the most reliable methods of translating evidence into statements to guide practice, but are typically not designed to support shared decision-making. Decision aids represent widely advocated tools for shared decision-making [3]. Decision aids improve patients’ knowledge of options, their perception of feeling well-informed, and their clarity regarding what matters most to them [3].

Both guidelines and decision aids face similar challenges: their production and updating is highly resource-demanding, they are often not based on best available evidence, they may be hard to find and use, and their uptake is highly variable in practice [4]. We have previously reported how we have addressed these overarching challenges in the Sharing Evidence to Inform Treatment decisions (SHARE-IT) project [4]. SHARE-IT has resulted in a new generation of generic decision aids linked to trustworthy guidelines and evidence summaries in digitally structured formats [4, 5]. These encounter decision aids are designed to be used by clinicians and patients to explore together the management options and facilitate shared decision-making [4].

We report here our detailed approach to SHARE-IT encounter decision aids conceptual and technical development, and results from iterative user-testing to achieve user-friendly presentation formats. We also report how these encounter decision aids were integrated in MAGICapp, a digital authoring and publication platform for guidelines and evidence summaries. In MAGICapp, the evidence data is structured in a way that enables a semi-automated production of decision aids, and facilitate dissemination and dynamic updating of them, within the context of guidelines [4].

## Methods

### Overview and rationale

**Fig. 1:**
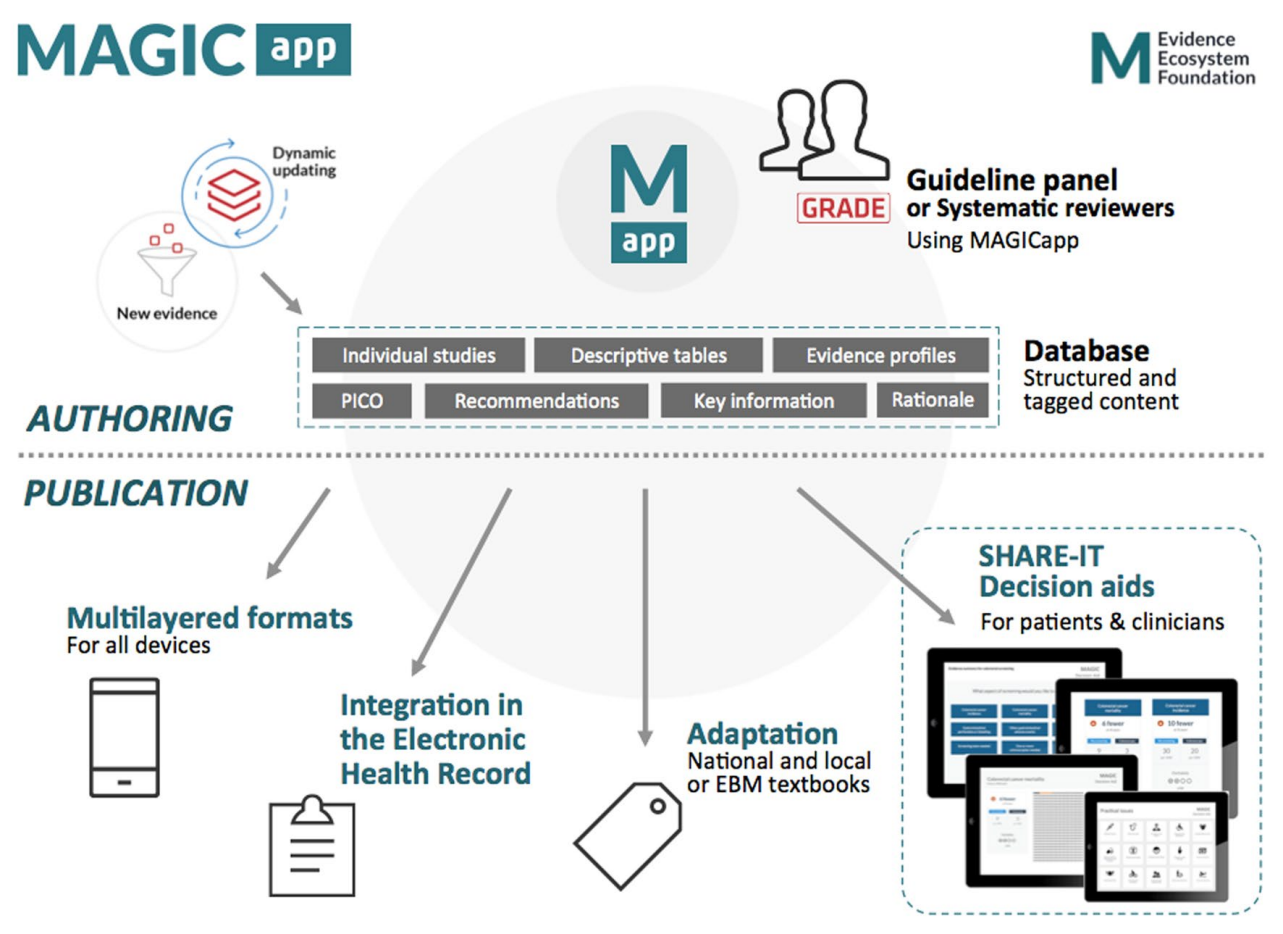
Generation of SHARE-IT encounter decision aids through the MAGICapp authoring and publication platform

SHARE-IT was initiated in 2012 by the non-profit MAGIC Evidence Ecosystem Foundation [6]. Combining research with innovation and product development within a digital and trustworthy evidence ecosystem, MAGIC aims to provide clinicians and patients with user-friendly tools for decision support implemented at the point of care [4, 5]. Its online authoring and publication platform—the MAGICapp (Fig. 1)—was initially developed to apply GRADE methodology (Grading of Recommendations Assessment, Development and Evaluation) [7] to author, publish and dynamically update trustworthy guidelines in user-friendly formats [5]. We quickly identified the need to translate digitally structured data into tools that could support shared decision-making in the clinical encounter.

We conceived SHARE-IT in collaboration with the DECIDE project (Developing and Evaluating Communication strategies to support Informed Decisions and practice based on Evidence), a multi-national research project initiated by the GRADE working group and funded by the European Union [8–11]. After the DECIDE project ended in 2014, our team continued user-testing and developing the decision aids. A major consequent refinement was the addition of a display of practical issues to complement evidence on benefits and harms [12, 13].

**Fig. 2:**
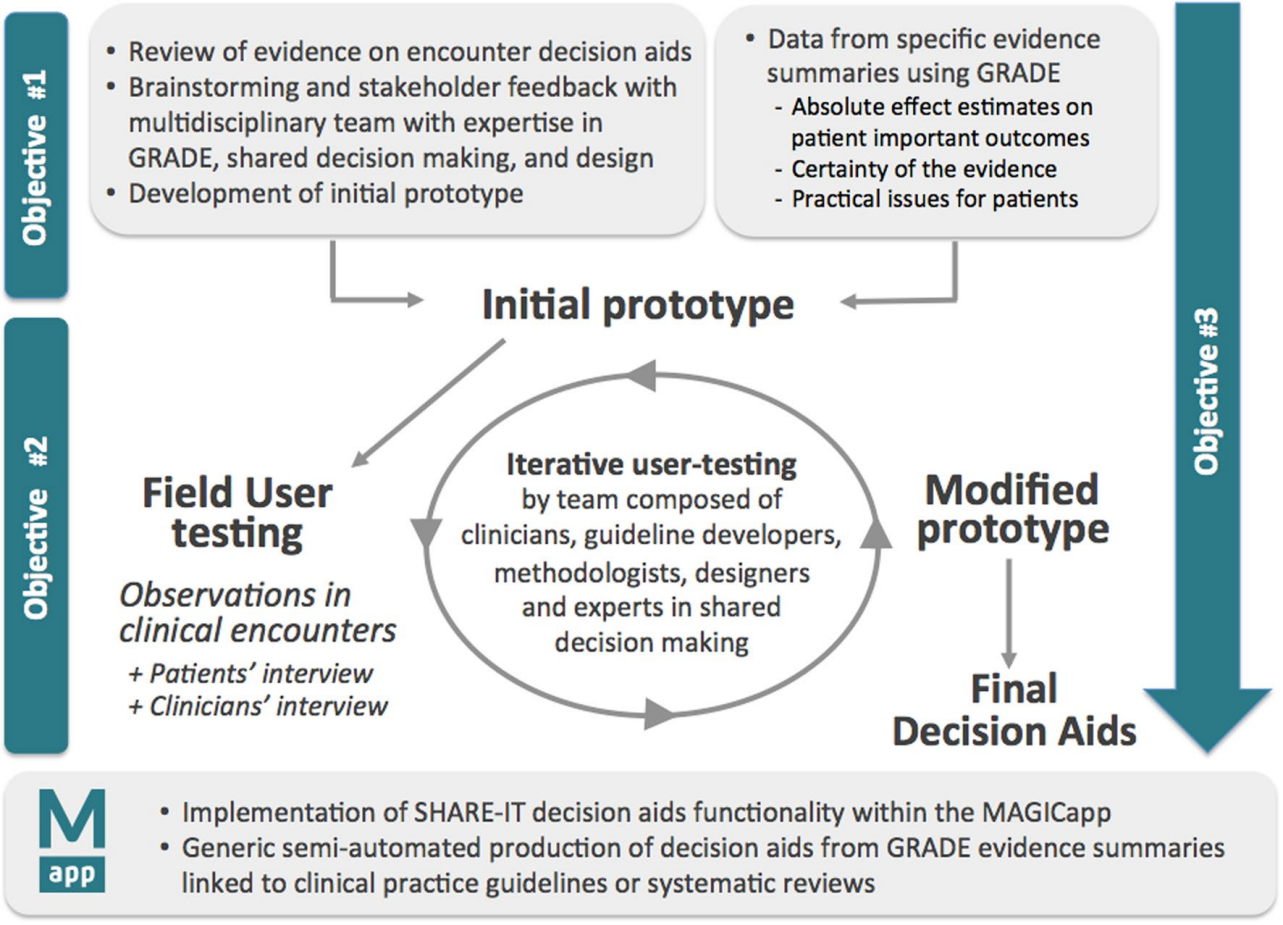
Prototyping, User-testing and Implementation of SHARE-IT decision aids in the MAGICapp for their generic production

Based on initial feedback from experts and stakeholders, principles of human-centred design were applied, and led to iterative revisions of the encounter decision aids through repeated observations with patients and clinicians engaged in real-life decision-making [14]. Figure 2 shows the three phases of our project, as defined in DECIDE: (1) brainstorming and stakeholder feedback with a multidisciplinary team to develop a conceptual framework and prototype decision aid; (2) iterative development and user testing of the decision aids; (3) their generic semi-automated production, from GRADE evidence summaries linked to guidelines, in MAGICapp.

### Development of the decision aids

#### Sketching the initial template

We based our initial prototype on evidence regarding optimal formats for shared decision-making, with a particular focus on encounter decision aids. In particular, our template was inspired by decision aid cards centred on key outcomes and issues meaningful to patients pioneered by Dr. Montori and his team in the Mayo Clinic Knowledge and Evaluation Research Unit [15].

Our team combining expertise in GRADE methodology, shared decision-making and human-centered design, built several prototypes with the help of an interaction designer (FA). We followed a modified “mobile first” approach [16] in sketching and creating the initial template, using an online calculator [17] and Blueprint software [18] which allowed us to quickly customize and test our prototypes on tablet screens. We judged use of the tool on a desktop computer would not optimally facilitate face to face communication between patient and clinician in clinical encounters.

#### Stakeholder feedback and brainstorming on the next iterations

To move from the initial template to a conceptual framework and prototype decision aids linked to guidelines, we conducted three face-to-face meetings with stakeholders in DECIDE (Canada 2012, Italy 2013, and Peru 2013) [8]. The meetings involved clinicians and experts in shared decision-making, guideline development and designers. The experts evaluated the initial template and subsequent prototype decision aids and participated in brainstorming, discussion and feedback.

### User testing

Following stakeholder feedback, the team prepared the prototype for formal user testing in clinical encounters to learn about the design from a user’s perspective to improve its next iteration as opposed to developers or experts [19].

#### Materials and setting

Prototype encounter decision aids were built for a variety of clinical scenarios, including 21 decisions concerning antithrombotic therapy and one for cancer treatment [20, 21]. The choice of supporting evidence summaries was driven by the fact that several authors had conducted extensive GRADE evidence summaries related to an update of the American College of Chest Physicians Evidence-Based Clinical Practice Guidelines on the topics [20]. Antithrombotic therapy decisions addressed new oral anticoagulants (for pulmonary embolisms, deep vein thrombosis and atrial fibrillation) and thromboprophylaxis during pregnancy. We used GRADE evidence summaries published in digitally structured formats in MAGICapp [4, 5]. The cancer scenario addressed adjuvant tamoxifen treatment to prevent recurrence of breast cancer; we produced a GRADE evidence summary based on trial results [15]. All decision aids reflected decisions deemed particularly sensitive to patient values and preferences, typically accompanying weak recommendations according to the GRADE framework [22]. The decision aids were available in English and Norwegian.

The completed Standards for Reporting Qualitative Research checklist is included as Additional file 1.

#### Participants and recruitment

We performed user-testing of the decision aids in real-life consultations in secondary and tertiary health care facilities in Norway (Innlandet Hospital Trust, Gjøvik and Oslo University Hospital, Oslo), the United Kingdom (Ninewells Hospital, Dundee) and Canada (McMaster University Hospital and Hamilton General hospital, Ontario). A convenience sample of physicians was recruited, with variable experience in risk communication and variable familiarity of the clinical topic. Patients were recruited through the participating physicians as part of either their outpatient clinic visits or acute hospital inpatient admissions.

#### Data collection

A team member provided a brief demonstration of the tool, typically less than 10 min, demonstrating to participating physicians the use of the encounter decision aid. A study member directly observed the clinical encounter, noting the use of the decision aids, and patients’ questions regarding their management. We audio-recorded and transcribed the consultations, followed by professional translation to English for encounters in Norwegian.

Directly after the consultation, the team member who had observed the encounter conducted separate think-aloud sessions with patients and clinicians. We used a semi-structured interview guide with questions eliciting feedback on their experience and on the format and usability on the decision aid. The focus of our attention was their actual experience. Suggestions for improvement were also collected. At the end of the interview respondents completed the 20-item COMRADE instrument, which provides a quantitative assessment of *risk communication* and *confidence in the decision* [23]*.* COMRADE uses a 5-point scale from 1(strongly agree) to 5 (strongly disagree) [24].

#### Data analysis

**Fig. 3:**
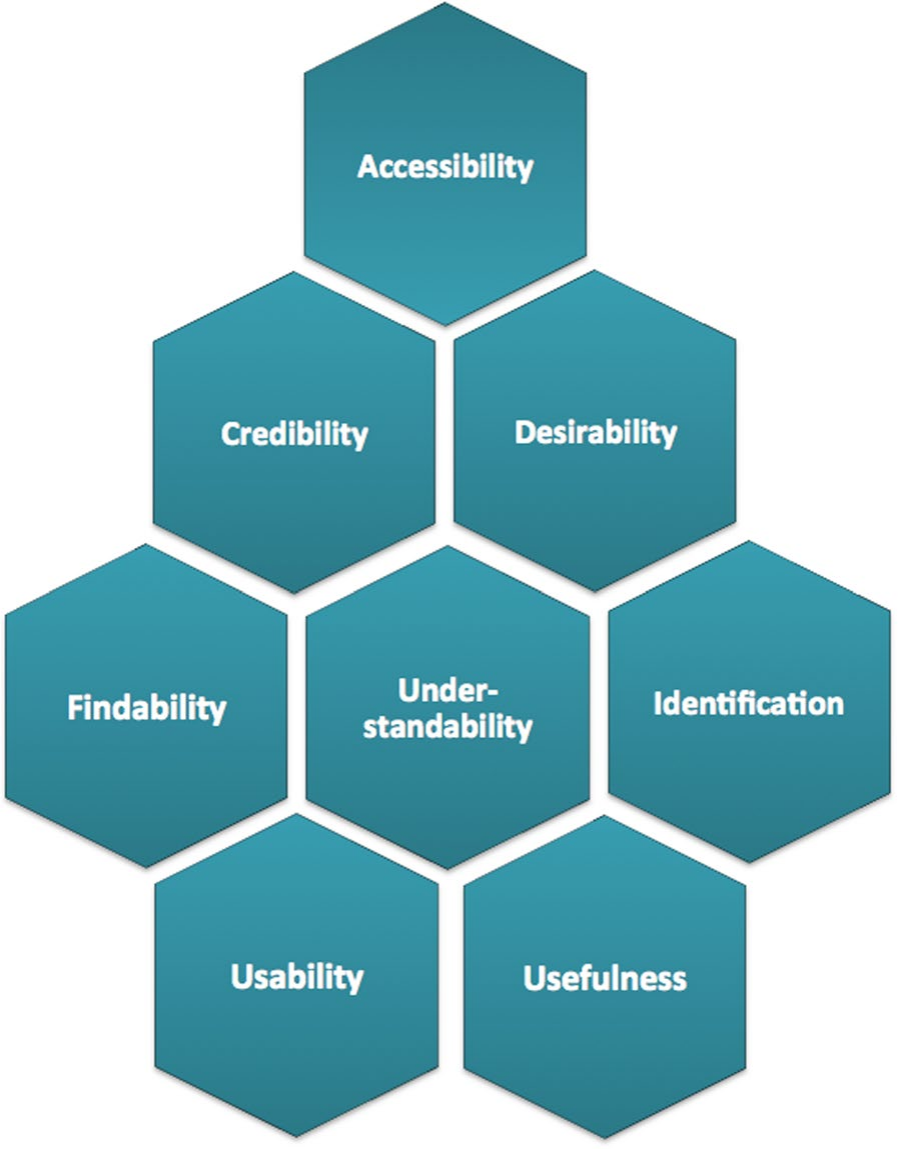
Modified Morville’s model for testing the experience of users

We coded transcriptions of the audio-recordings of the clinical encounters and semi-structured interviews. Content analysis was performed through both deductive and inductive approaches, searching for units of meaning and condensing text [25]. We then compared and added codes to the results and searched for barriers, problems and facilitating elements or characteristics of the tool that influenced the user experience and the process of shared decision-making. Each element of meaning was coded using a revised version of Morville’s framework (Fig. 3) categorizing eight different facets of “user experience” to sort results into categories: findability, usefulness, usability, understandability, credibility, desirability, identification and accessibility [26, 27]. Finally, each element was also coded with regards to the quality of the experience—i.e., positive feedback, neutral experience, suggestions for improvement of the tool, minor frustration and major frustration (“show stoppers”).

## Results

### Development of framework and prototype encounter decision aids

**Fig. 4:**
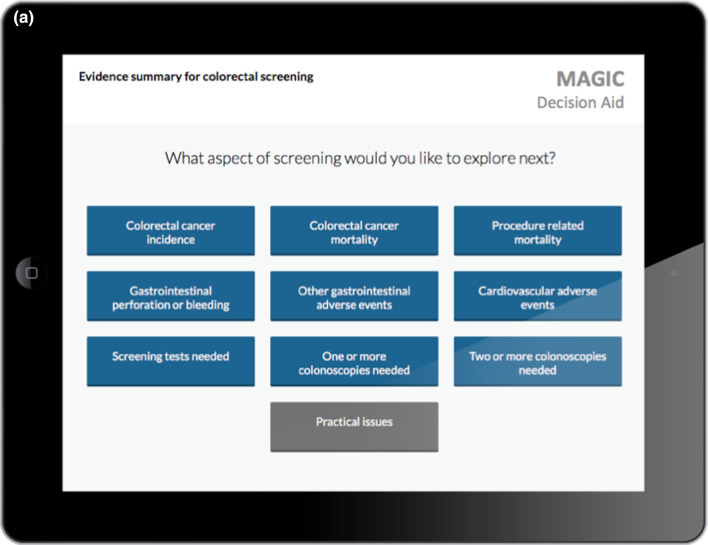

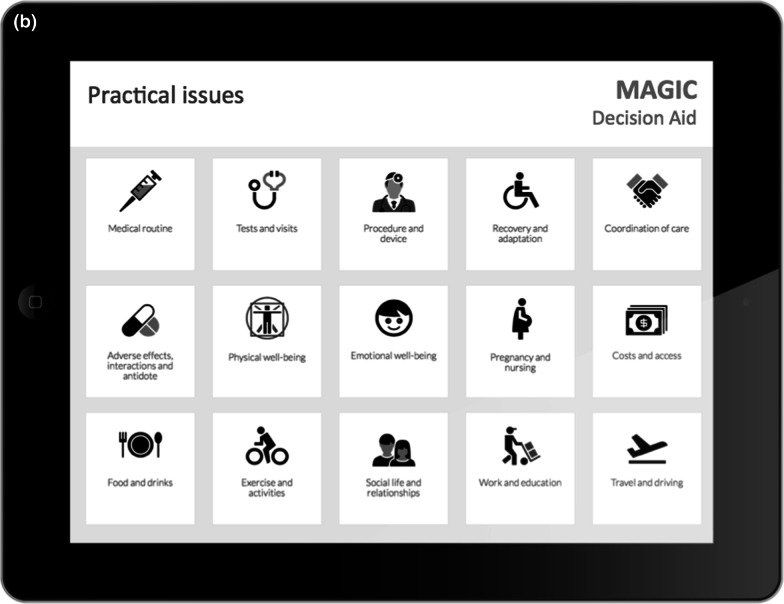
Example of encounters decision aids; **a** first layer displaying outcomes and practical issues relevant to a given decision; **b** underlying layer for the exploration of practical issues

**Fig. 5:**
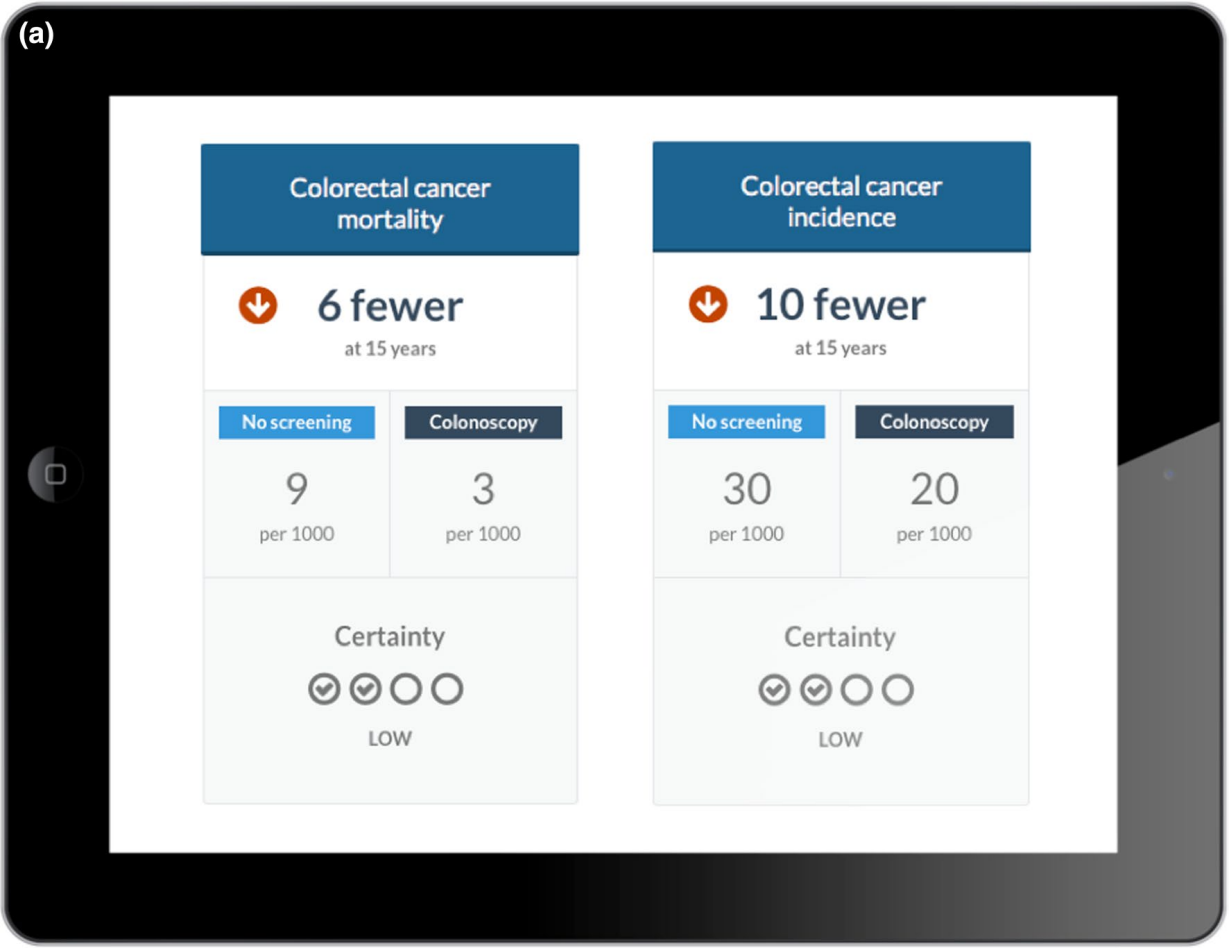

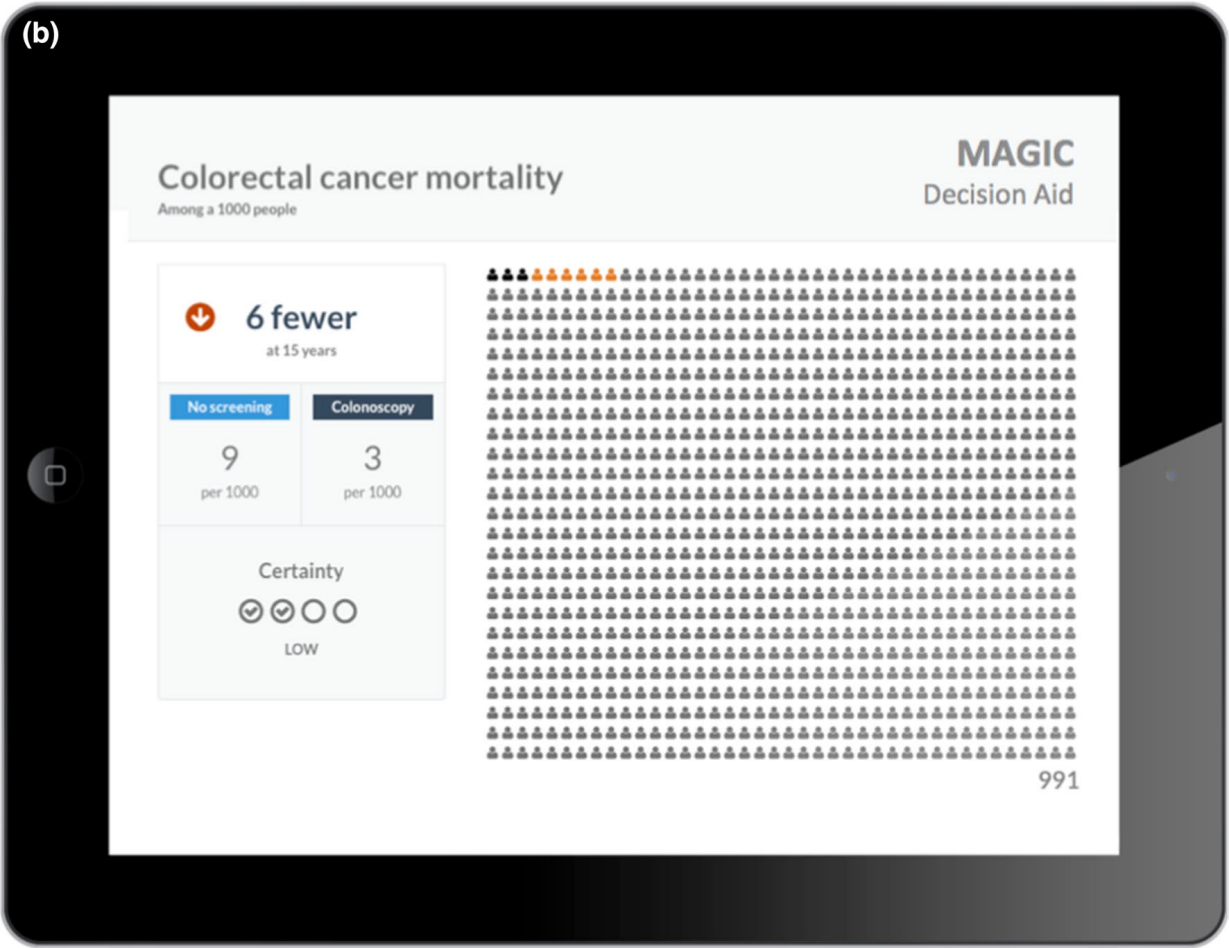
Example of encounter decision aids; **a** Second layer comparing outcome cards; **b** third layer for further discussion of absolute risks and certainty

In the three DECIDE stakeholder meetings, 22 experts provided extensive feedback and suggestions to inform the conceptual framework and prototype decision aid formats. Core desirable features of the decision aids included: (1) communicating risk and uncertainty, (2) navigating the content, (3) facilitating use of the encounter decision aids both within and outside the clinical encounter, and (4) the inclusion of burden of treatment/practical issues. Following several iterations, the experts reached consensus on a prototype decision aid ready to undergo user-testing (Figs. 4, 5).

### Iterative development through user testing

We performed four major iterations of the decision aid presentation formats, based on the observations and analyses of 28 real-life consultations with physicians and patients (median age 53, range 19–90, 64% women). Participants used tablet computers (e.g., iPads) in 47% of the consultations, desktop or laptop computers in the remainder. COMRADE response rate was 72.7% (n = 20). Patients rated both items of risk communication and items in their confidence in the decision with a median of 1 on the 5-point scale (i.e. “s*trongly agree*”).

#### Overview of user-experiences

Table 1 provides a quantitative summary of user-experiences with the encounter decision aids categorized according to the revised Morville’s facets (Fig. 3) and the quality of the experience coded as: positive feedback, neutral experience, suggestions for improvement, minor frustration and major frustration. These were based on content analysis of transcripts of the consultations and semi-structured interviews. Elements of major or minor frustration, with or without suggestions for improvement, were the main drive for improvement of the tool across iterations, as they affected most the user experience. Neutral experience referred to statements voiced by users, which were neither positive nor negative, that provided insight on how they navigated across the different features or functionalities of the tool. Together with spontaneous positive feedback, they pointed at functionalities of the tools that worked smoothly in the course of the clinical encounter.

**Table 1:** Quantitative summary of facets of user-experiences and the quality of the experience using the decision aid

Facets of user-experience	Quality of the experience					
	Neutral experience	Positive feedback	Suggestions to improve	Minor frustrations	Major frustrations	Total
Accessibility	3	1	16	20	2	42
Credibility	1	6	1		–	8
Desirability	11	30	9	4	–	54
Findability	–	–	1	–	–	1
Identification	29	2	1	–	–	32
Understandability	126	34	11	11	6	188
Usability	43	33	14	26	2	118
Usefulness	40	81	17	5		143
Total	253	187	70	66	10	586

We coded 586 observed units of meaning across all interactions. Most reported issues involved understandability and usefulness, whereas findability and credibility aspects were least reported. Regarding the quality of the experience, there were no showstoppers. The majority of observations (43%) related to ways to use the tool in consultations, while 32% were expressions of positive feedback (e.g. praise, elements of delighted surprise), and 12% suggestions for improvement. We provide below a synthesis of the findings in each of the facets, with illustrative quotes directly from the consultations and interviews.

#### Accessibility

Across iterations, the majority of comments concerned the readability, font colours or size, or visual contrast (e.g. they needed to put on their glasses) with other expressions of aesthetic preferences. Patients perceived the tools worked well for themselves, but speculated on how it may not be as accessible for others, such as colour-blind people, or older patients who may be more averse to technology:

57-year-old man with venous pulmonary embolism: *“If you were using this tool with other people, other than me, just people 65/70 years old and afraid of the new technology, the picture would be a little blurred.”*

46-year-old woman with breast cancer: *“Being faced with an iPad or a laptop may put off some older women”*

#### Usability

The majority of users, both clinicians and patients, reported that the tool was easy and simple to use without need for explanation, with a design that supported usability.

Clinician: *“Actually, it's quite self-explanatory, really, the whole app.”*

Clinician: *“Everything was presented in a very neutral way. That is, no scary fonts, no green or red colours that might imply certain values. I felt everything was easy to read and interpret”*

Physicians integrated the tool in their work-flow and conversations using expressions such as “let’s go back and see”, pointing at outcomes on the screen, asking what the patient wanted to look at first or leaving the direction of the conversation to the patient. Several did this together with the patient, describing the numbers, using the tablet together and the tool engaged both patients and clinicians:

Clinician: *“So what do you think we should do with, what's most important for you do you think, when to choose a medicine?”*

Clinician: *“Do you want to talk about the risk of bleeding first or the risk of clotting first or the practical considerations?”*

Two clinicians commented that it took some time to get used to the tool and get it fully integrated in their consultation, or struggling with finding the appropriate language:

*Clinician*: *“Quite honestly, I felt a bit awkward using the tool, but it was my first time using it. Like any new tool, I am sure it takes practice to make it flow smoother.”*

*Clinician*: *“I thought the tool was a great idea. It was a little harder to come up with the language to use to discuss it with the patient than I had expected, but in general, I thought it worked well.”*

#### Understandability

Patients and clinicians used their own vocabulary to express how they understood how the tool could help them individualise the conversation related to risks, value elicitation and uncertainty in decision-making.

Clinician: *This tool is supposed to help me explain, to compare the two [options] to help you decide what you would like to do at this point.”*

Clinician: *“We can reduce that number by 58 people [per 1000] if we give Rivaroxaban. So there is some value about taking it but there are some downsides. Now what’s the downside you are worrying about most?”*

Visualisation of the evidence in the tool was informative, clear and easy to understand for most participants, while one patient reported that the pictographs were confusing:

76-year-old man with venous thromboembolism: *“I liked the way it was presented […] both numbers and figures were easy to understand”*

*33-year-old pregnant patient with increased risk of venous thromboembolism: “Confusing looking at the board with figures [i.e. pictographs]”*

All consultations contained discussion about absolute risks of different outcomes. This part of the conversation using the tool was mostly led by the clinician. There was a broad variability in how clinicians and patients rephrased the risk estimates (e.g. “small” or “high” and also applying it to the specific patients’ situation, particularly when less applicable:

Clinician: *“I wish I had data to say: okay if we had 1000 guys who rode a motorcycle all the time, what’s the risk. I don’t have that, and I will never be able to get that.”*

Clinician: *“I am not going to tell you can’t play but I’m going to tell you, you have to be comfortable with carrying that risk. I don’t want you to play* [sport] *scared right. You know, the other thing we talked about briefly is what happens if you bleed on Rivaroxaban, you know if somebody jabbed you or something you are going to bruise up.”*

In the first iteration of the decision aid prototype, the certainty of the evidence was labelled as “confidence” without any further information.

33-year-old pregnant patient with increased risk of venous thromboembolism giving feedback on the decision aid: *“The box where it said something about confidence in the results, it said low or high. It could explain if it was the medical confidence in the results or the users' confidence in the results.”*

We then systematically incorporated in later iterations the main reason for the degree of certainty, taken from GRADE summary of findings. Most clinicians still often ignored certainty, except in specific conversations discussing mortality when faced with uncertainty, as they perceived patients would struggle to understand it:

*Clinician: “I am not quite convinced that “uncertainty” is a concept that patients can grasp or that the way it is presented in the tool is all that helpful.”*

*Clinician*: *“I did appreciate having the quality of the evidence accessible as well. Though I don’t recall using this feature more than one or two times in the encounter, it was nice to know that it was there.”*

¾Users reported that medical abbreviations were not understandable, and the generic drug names needed explanation.

Clinician*: “The only thing maybe ... one must always explain this with VTE, which is abbreviated.”*

#### Usefulness

The majority of patients and physicians perceived that the tool was useful and supported better information, value clarification and shared decision-making. They felt the tool contributed to reaching a decision together, although some highlighted decision aids were not necessary to achieve a good consultation.

*Clinician: “The patient thought that the tool made the benefits of her decision to continue taking tamoxifen the 10 years clearer; she had been told there was a benefit but did not know how much of a benefit before today.”*

After several consultations, both physicians and patients noted that they had been somehow surprised by the decision made. It allowed the presentation of useful information that would otherwise not have been brought up in the conversation:

*73-year-oild man with venous thromboembolism: “Yes, if the tool wasn’t used, I would probably not have gotten the information.”*

*Clinician*: *“Surprisingly, the patient ended up choosing to stop medication after 3 months, congruent with his values. [It] probably wouldn’t have been the mother’s or father’s decision. [They] would have preferred that he stopped basketball for this health”.*

*Clinician: “At the time of consent, we were convinced using the tool wouldn’t change anything. After the consultation, we thought it was really useful to look at the evidence, in particular graphically.”*

Another key observation related to the use of the tool, particularly with tablet computers, was that physicians and patients shifted posture from sitting across each other to side-by-side, looking at the tablet, and even holding it together when having a conversation.

*64-year-old man with venous thromboembolism reflecting on the use of the decision aid: “It shown you graphs […] rather than just sitting back verbally across the desk and saying… like Dr. X did.”*

The simplicity of the various presentation formats, allowing an overview, the comparison of benefits and harms, and the exploration of the same information in different formats, both visually and numerically, was highlighted as particularly useful:

*52-year-old woman with breast cancer giving feedback on the decision aid: “The simplicity of it is actually one of its strengths I think”*

*64-year-old man with venous thromboembolism reflecting on the use of the decision aid: “I think a picture says 1000 words […] it’s giving you the stats, it’s also showing you stats. […] I think for many people it’s easier to understand that when you see the graph than just to hear it and just see a number.”*

Users appreciated the possibility to easily compare and switch between different clinical outcomes, supporting the natural flow of the conversation rather than following a pre-defined pathway:

*52-year-old woman with breast cancer giving feedback on the decision aid: “The most helpful feature is the flexibility - being able to switch between different clinical outcomes for any given clinical scenario. […] I like the fact that you can bring them up side-by-side as well, I think that's really helpful rather than kind of exiting and entering, you know, and trying to remember the ones from before.”*

*Clinician: “If the conversation shifts in a particular direction, e.g., the patient wants to talk more about bleeding, we can shift the tool in that direction. I really appreciated this flexibility, because it made my discussion more responsive and natural.”*

Views around the overall amount of information available or displayed were highly variable:

*53-year-old woman with venous thromboembolism reflecting on the content of the decision aid: “A lot of information. Should not be less but is difficult to grasp all of it.”*

*71-year-old man with venous thromboembolism reflecting on the content of the decision aid: Patient 15 (VTE): “No superfluous information. Very short and concise so rather have some more details.”*

Decision aids varied in the total number of outcomes that they included (i.e. up to 10 outcomes), which led to variable feedback on their optimal number or the order in which they may be presented at the top level of the tool, although users also recognized the value of choosing which one to focus on in the clinical conversation:

*Clinician: “Actually, [the outcomes] are lost, the really important ones […] perhaps a bit overwhelming.”*

*Clinician: “I was going to say if there's a way of having the ones that are actually more relevant, but the point is that it's what the patient thinks is relevant isn't it?”*

This issue led us to develop an authoring feature in MAGICapp that allows the selection of which outcomes of the GRADE summary of findings table to display in the decision aids, and the possibility of relabelling the outcomes (Table 2).

**Table 2:** User-testing findings of barriers and issues and solutions to inform iterations of the encounter decision aids

	Barriers and issues discovered during the user testing	Changes in the subsequent iterations
Accessibility	Lack of contrast in text and pictographs Scrolling was needed to see all content when tablet was vertical Wi-Fi issues in hospitals	Enhanced contrasts, changed colours Scrolling removed Created off-line version and print version
Usability	Suggestion of a top layer to ease the introduction to the tool Difficulty coming up with language to use the tool Suggestion to combine the tool with information provided to patient before encounter Suggestion to have the possibility to change the denominator in the icons (and possibly in the numbers)	Supportive sentence “What aspect would you like to discuss next? Choose and compare” outcomes to raise choice awareness Possibility to change data entry and display directly in MAGICapp feeding in the interactive decision aid content
Understandability	Concept of certainty Medical abbreviations difficult to understand Generic drugs names confusing	Main reason for uncertainty made available one click away Names and descriptions of outcomes can be edited
Usefulness	Great variability in the perception of the appropriate amount of information, in particular the number and order of outcomes Useful to have something to bring home Suggestion of a feature that could compare several options	Number of outcomes and their order can be selected independently of underlying evidence profile Print version developed Multiple comparisons prototype in development
Identification	The patient’s risk might be different from what is shown in the tool	Highlight during demonstration and in quick educational modules that this is encounter decision aid to be used together with a clinician, who can adapt content to each patient, highlighting potential similarities or differences
Credibility	Different colour of outcome card for practical issues could lead to selection bias	Specific design developed to display practical issues and navigate across them [12]
Findability	Clinician needed more information on evidence behind estimates in decision aids	Integration with MAGICapp with decision aids directly linked to GRADE evidence summaries

Finally, several patients highlighted that it would be useful to have written information to bring home to be able to remember the content of the conversation and to discuss with close ones:

47-year-old man with venous thromboembolism: *“So, something that complements this that you can look on your own, at home, that’s interesting. And spend a little extra time looking at it. Because you know, I’m going to go home, and my wife is going to ask me 100 questions.”*

This issue led to the development of a printable version of the decision aid (Table 2).

#### Identification

The patients identified with the content and felt the tool was about their own choice. Physicians used the tool to enhance awareness of choices or to find out what mattered most, for example steering the conversation towards the daily life implications for patients:

90-year-old woman with venous thromboembolism: *“So I just need to be careful not to prick myself with the needle when I’m sewing”*

Some patients felt that the physicians’ knowledge was more relevant to their own decision than what was presented in the tool. Clinicians also spontaneously clarified when the patient’s risk might differ from what is shown:

*Clinician*: *Now this data, this stuff that we constructed from big studies, but this is a little different from you.”*

#### Credibility

Both the physicians and patients perceived the tool as trustworthy, both in content and the way it was presented:

47-year-old man with venous thromboembolism reflecting on the decision aid: *“I feel confident I saw all important information to take a decision”*

Clinician: *“The order was correct: why you take the medication, what prevents it, the most important complications”*

#### Desirability

Many clinicians and patients expressed a preference in having the tool used in a consultation, rather than not, and one patient thought that the tool would empower patients.

74-year-old man with venous thromboembolism reflecting on the use of the decision aid: *“I feel a bit privileged coming here, cause other patients that go to their GP might not get the same introduction”*

*Clinician: “I think that a problem with many of these sorts of decision aids they just get too complicated, so I think this is quite nice” … “I think it's great, I, I'd like to be able to use it in the clinic actually, because I think it's quite, quite a helpful way of practically explaining things to people with, with some detail, but not too much detail”*

#### Findability

Since the decision aid was directly provided for each encounter, we were unable to explore issues related to how challenging it would be to find it during an encounter. The only aspect that came up related to physicians’ needs to have easy access to the supporting evidence for the estimates-effect provided in the decision aid. This issue was solved by the integration of decision aids in MAGICapp where all underlying evidence is directly linked to the decision aid (Table 3).

**Table 3:** Main concepts and features of the decision aids

- Electronic generic framework for decision aids integrated in an authoring and publication platform for guidelines and evidence summaries (MAGICapp)
- Decision aids are semi-automatically produced and updated based on content in MAGICapp with adaptation possibilities (e.g. wording and number of outcomes, language)
- Multi-layered presentation format: ○ First layer displays the list of patient-important outcomes and practical issues (Fig. 4a) ○ Second layer displays interactive outcome cards with evidence estimates, certainty, and patient-important practical issues across 15 generic categories. Possibility to interactively compare two or more outcomes in parallel (Fig. 5a) ○ Third layer displays a corresponding set of pictographs showing the absolute risk with each option (Fig. 5b) and practical issues related to the treatment option (Fig. 4b)
- Educational module developed http://magicproject.org/161128/ and integrated in MAGICapp. Content was generated to mimic the very short demonstration used during user-testing
- Print functionality of decision aids create pdf files that can be printed or used for notetaking and/or to bring home
- Prototype for comparisons between multiple options are developed and implemented in a BMJ Rapid Recommendation [28]
- Offline feature so decision aids can be used without use of Internet
- Widgets from MAGICapp to grab and show a given decision aid on any other online platform Example: Rapid Recommendation on Prostate cancer screening (https://www.bmj.com/content/362/bmj.k3581 to BMJ infographic) which links to MAGICapp content, including widgets to decision aids for various profile of patients

### Changes made in presentation formats across iterations of the prototype

We performed four major iterations of the decision aid presentation formats based on user-experiences. Table 2 summarizes the identified issues and barriers followed by specific solutions that were implemented across iterations. Final versions of the generic decision aids were reached after the team reached consensus that the decision aid prototype successfully involved patients in shared decision-making and satisfied the needs of patients and physicians.

A final version of the generic decision aids was reached and read for integration in an authoring and publication platform for their generic and semi-automated creation. Table 3 summarized the main features in the decision aids.

### Integration in MagicApp

We integrated the prototype in MAGICapp (Figs. 4, 5) The technical integration of the final version of the decision aid prototype specifically resulted in a: (1) automatically generated decision aids for all available GRADE evidence summaries linked to recommendations in the platform, (2) access to all underlying evidence, (3) automatic update of decision aids when the evidence summary is updated and (4) selecting the number of displayed outcomes and changing labels for more lay language wording whenever relevant.

MAGICapp has numerous (> 1000) available decision aids. Since users and customers of the platform are responsible for producing the evidence summary and own it, we have not performed a formal quality assurance of accuracy and clinical relevance of all available decision aids. The integration in MAGICapp also makes it possible to easily generate widgets so the decision aids can be integrated on other online platforms (e.g. button links to decision aids from the BMJ Rapid Recommendations.)

## Discussion

We have developed encounter decision aids linked to evidence summaries that have informed trustworthy guidelines to facilitate shared decision-making with patients at the point of care. User-testing in real clinical encounters revealed opportunities for improvement in readability, understandability, usability and information overload that we addressed through four design iterations. After addressing these issues, user-testing demonstrated that the developed decision aids are understandable and intuitive; support conversation on issues that matter most to patients; and help clinicians share evidence regarding benefits, harms, their associated degree of certainty, along with practical issues relevant to each management option.

### Strengths and limitations

Strengths of our project include the user-testing of the decision aids in real-life consultations and in a variety of clinical settings. Suggestions for improvements from users resulted in changes that produced a higher degree of usability and accessibility.

The brief introduction to the tool proved sufficient that clinicians and patients described it as easy to use and understand.

A key element is the perceived trustworthiness of the content, which was captured by the user experience dimensions of credibility and identification. Clinicians also outlined their need to link back to the detailed evidence summary and sources of uncertainty, which the tool provides.

In regard to limitations, our study may have selected clinicians who were more versed in, and more enthusiastic about innovative approaches for risk communication. Moreover, the current study focused only on situations in which patients face two management alternatives and did not explore decision aids for multiple comparisons. Development a tool dealing with multiple options is in progress, and is currently included in recent BMJ Rapid Recommendations, for example on screening for colorectal cancer [28].

Shared decision-making hinges on clinicians having access to up-to-date and quality appraised evidence [29]. This was achieved by integrating the framework in MAGICapp to semi-automatically produce decision aids based on content from guidelines and evidence summaries. This, however, requires someone to carry out the updating process, which remains a hit-or-miss phenomenon.

User-testing was performed before mandatory social-distancing required by COVID-19 restrictions. Generalizability to virtual consultations remains to be confirmed, although the online nature of the tool allows its use from afar.

### Implications for encounter decision aid production

Information overload is a critical challenge in the development of evidence-based tools. This is particularly true for decision aids, which risk excessive information that may compromise useful conversations. To that end, the design of our generic decision aids was heavily inspired by the work of Montori and colleagues who identified the need for encounter decision aids to be as “quiet” as possible: i.e. that the tool does not impose a necessary sequence of predefined algorithms of questions and answers, that pushes the interaction into a pre-defined script, but instead organizes information so as to support the actual conversation that occurs between clinicians and their patients on what matter most to them [15, 30].

We implemented a similar approach through our interactive multi-layered formats. User testing allowed us to explore those elements that were better to highlight in top levels and those that could be presented in deeper layers. The final version of the decision aids has a top layer displaying only the list of outcomes and practical issues, without any numbers. Intermediate layers provide a synoptic view of each potential benefit or harm, followed by deeper layers providing detailed pictographs and underlying information, such as reasons for uncertainty in the estimates of benefits and harms.

Such information was sometimes useful and other times distracting. Iterative user-testing demonstrated that patients appreciated the flexibility of this approach, as well as the possibility to easily switch between different outcomes and issues. Moreover, as the number and labelling of outcomes in the decision aids sometimes needed to differ from the supporting GRADE evidence summary, we implemented the functionality to edit the decision aids automatically generated in MAGICapp.

The multi-layered approach allows the display of more outcomes than static GRADE summary of findings tables allow (usually not more than 7 most critical and important outcomes) [31]. In common with other encounter tools tested by Montori et al., patients reached most decisions after exploring only a selection of outcomes (usually 3 to 4) [15, 30].

SHARE-IT represents the first successful, user-tested effort to fully integrate production of decision aids with the production and dissemination of evidence summaries, recommendations and guidelines. This integration also makes it possible to adapt the content (e.g. to national guidelines or policies or certain populations). The content and quality of the decision aids are, however, dependent on the quality of the evidence summaries.

Education and training are also central in any implementation strategy. Use of SHARE-IT decision aids required minimal demonstration of the tool, as shown in our short online education module [32]. This was sufficient to explore it intuitively during a real clinical encounter. As piloted by several clinical educators in our team, example of such decision aids linked to guidelines can be used in rounds and bedside teaching, a strategy that warrants further evaluation. This may help to overcome an important barrier: the benefits of using decision aids (as well as engaging in shared decision-making altogether) are really known after one has experienced it.

## Conclusion

Our study provides a proof of concept that encounter decision aids can be generically produced from GRADE evidence summaries or recommendations for clinical practice. Further evaluation is needed in more clinical contexts and as part of educational and broader implementation strategies. This would require that decision aids are available for a large number of clinical decisions. The integration of SHARE-IT decision aids in MAGICapp offers great potential in scaling up their production and continuous update along with evidence summaries and clinical practice guidelines.

## Supplementary Information

**Additional file 1.** Standards for Reporting Qualitative Research checklist.

## Data Availability

The datasets used and/or analysed during the current study available from the corresponding author on reasonable request.

## Abbreviations

DECIDE: Developing and Evaluating Communication strategies to support Informed Decisions and practice based on Evidence project
IPDAS: International Patient Decision Aid Standards
GRADE: Grading of Recommendations Assessment, Development and Evaluation
SHARE-IT: Sharing Evidence to Inform Treatment decisions project
